# A method to detect discontinuities in census data

**DOI:** 10.1002/ece3.4297

**Published:** 2018-09-20

**Authors:** Chris Barichievy, David G. Angeler, Tarsha Eason, Ahjond S. Garmestani, Kirsty L. Nash, Craig A. Stow, Shana Sundstrom, Craig R. Allen

**Affiliations:** ^1^ Zoological Society of London London UK; ^2^ Institute for Communities and Wildlife in Africa University of Cape Town Cape Town South Africa; ^3^ Department of Aquatic Sciences and Assessment Swedish University of Agricultural Sciences Uppsala Sweden; ^4^ U.S. Environmental Protection Agency Office of Research and Development National Risk Management Research Laboratory Cincinnati Ohio; ^5^ Centre for Marine Socioecology Hobart TAS Australia; ^6^ Institute for Marine and Antarctic Studies University of Tasmania Hobart TAS Australia; ^7^ NOAA Great Lakes Environmental Research Laboratory Ann Arbor Michigan; ^8^ School of Natural Resources University of Nebraska Lincoln Nebraska; ^9^ U.S. Geological Survey Nebraska Cooperative Fish and Wildlife Research Unit University of Nebraska Lincoln Nebraska

**Keywords:** discontinuities, discontinuity detector, ecosystem management, resilience

## Abstract

The distribution of pattern across scales has predictive power in the analysis of complex systems. Discontinuity approaches remain a fruitful avenue of research in the quest for quantitative measures of resilience because discontinuity analysis provides an objective means of identifying scales in complex systems and facilitates delineation of hierarchical patterns in processes, structure, and resources. However, current discontinuity methods have been considered too subjective, too complicated and opaque, or have become computationally obsolete; given the ubiquity of discontinuities in ecological and other complex systems, a simple and transparent method for detection is needed. In this study, we present a method to detect discontinuities in census data based on resampling of a neutral model and provide the R code used to run the analyses. This method has the potential for advancing basic and applied ecological research.

## 1 INTRODUCTION

**Figure 1:**
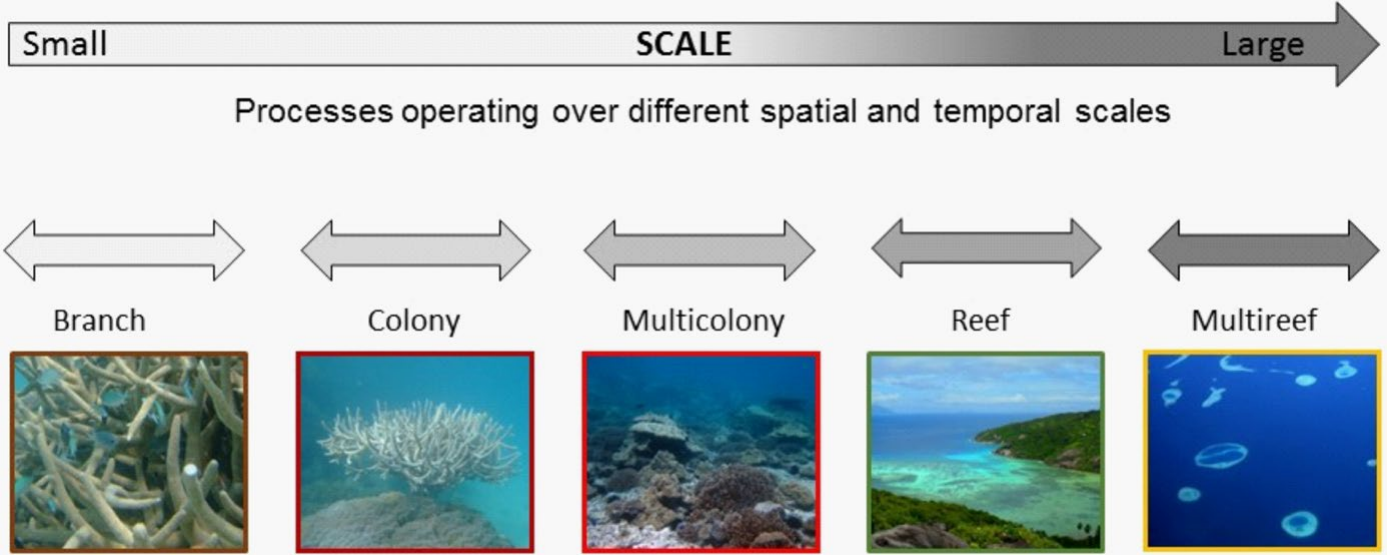
Processes occurring over different, discrete, spatial, and temporal scales and the resulting discontinuous distribution of physical habitat structure on a coral reef. Adapted from Nash, Allen, Angeler et al. (2014)

Complex systems are constantly adapting through interactions among components, with feedback loops that can generate system‐level behaviors that are not simply the sum of the parts (Levin, 1998). Accordingly, the dynamics of complex systems are difficult to predict using reductionist‐based models. Despite the variability within complex systems, there exist system‐level properties that are more stable than the individual components that comprise them. Coupled nonlinear interactions can create positive feedback loops over relatively discrete spatial and temporal scales. These “self‐reinforcing assembly states” (Stallins, 2006) are variously described as: “attractors” (Baas, 2002; Harrison, Massey, & Richards, 2006; Thompson et al., 2001), “stability domains” (Gunderson, 2000) or “domains of scale” (Wiens, 1989) or “scales of opportunity” (Garmestani, Allen, & Gunderson, 2009). Nevertheless, all of these terms refer to specific spatial or temporal windows within which pattern and process are tightly coupled relative to the other scales of the system. The result is a limited set of frequencies in both space and time at which dominant patterns and processes operate, reflected as the discontinuous distribution of pattern when compared across scales of analysis (Figure 1). Analyzing the dynamics of the cross‐scale pattern over space and time allows inference about changes in a system's relative resilience and other system properties (Allen & Holling, 2008).

Much of the discontinuity literature is based on the examination of body mass distributions because the scale at which an organism perceives the environment and procures resources is a function of its size (Calder, 1984). The body mass of an animal is an integrated measure of selective pressures affecting the evolution of a species and is highly allometric with many life history traits (Peters 1983). Organism body size affects speed and distance of travel (Harestad & Bunnel, 1979), processing of food, thermoregulation, physical structure, and the required quantity and aggregation of resources (Peterson, Allen, & Holling, 1998). Resources and habitat structure are not evenly distributed across the landscape; rather their availability varies among spatial and temporal scales (Wiens, 1989). Heterogeneously distributed resources across scales generate multimodal body size distributions, which is evidence of a discontinuity. Species are clustered at scales where resources are available and separated from neighboring body size aggregations by discontinuities corresponding to scales where resources are limited or highly variable in space and time (Nash, Allen, Angeler et al., 2014). Researchers have argued that discontinuities are a signature of hierarchical complex adaptive systems (Holling, 1992) and have been identified in ecological and other complex systems, including city and firm size distributions and economic data (Garmestani, Allen, & Bessey, 2005; Garmestani, Allen, Mittelstaedt, Stow, & Ward, 2006; Sundstrom, Angeler, Garmestani, García, & Allen, 2014).

Discontinuity research has direct application to ecosystem management (Angeler et al., 2016). Because discontinuity analyses objectively identify the scales at which pattern and process manifest, it is possible to examine the relationship between these scales and system features such as ecological resilience (Allen, Gunderson, & Johnson, 2005; Angeler, Allen, & Johnson, 2007; Baho et al., 2015; Stow, Allen, & Garmestani, 2007; Sundstrom, Allen, & Barichievy, 2012), invasion and extinction risk (Allen, 2006; Allen et al., 2005; Angeler et al., 2012; Raffaelli, Hardiman, Smart, Yamanaka, & White, 2016), and as an early warning signal of a regime shift (Spanbauer et al., 2016). For example, ecological resilience emerges in part from the distribution of ecological functions as provided by species. When a system has a high diversity of functions within each scale and a high redundancy of function across scales, it is better able to buffer disturbances (Peterson et al., 1998; Scheffer et al., 2015). Similarly, species with body sizes that place them close to a discontinuity are more likely to successfully invade a new ecosystem, or alternatively, are more likely to be driven to extinction (Allen, 2006; Allen et al., 2005; Angeler et al., 2012). Finally, identifying the proximity of a system to a regime shift is a fundamental goal in systems ecology, and it has been demonstrated that the number and location of discontinuities in community body size distributions are highly conservative through time, so significant shifts in the discontinuous distributions serve as an early warning signal of an impending system regime shift (Spanbauer et al., 2016).

### 1.1 Methods available for discontinuity analysis

Numerous methods have been used to identify discontinuities in datasets (Skillen & Maurer, 2008). In general, these methods compare the difference between adjacent ranked observations, for instance, the difference in body mass size between rank‐ordered species (Allen et al., 2005; Holling, 1992; Siemann & Brown, 1999; Stow et al., 2007). Early discontinuity research utilized a body mass difference index, in which differences between rank‐ordered body masses were compared, and a discontinuity was defined as a difference between two values that were greater than specified criterion. Relative measures were needed because direct comparisons between absolute values of ranked body masses are not meaningful. For example, a five‐gram difference between two shrew species is more meaningful than a five‐gram difference between deer species, simply because of the size of the animals, thus, a scaling exponent or log transformation was used. Another option was subjective transformations (Holling, 1992) in which bird body masses were transformed by 1.3 and mammals by 1.1, and a difference index was calculated as HI = (*M*_(*n*+1)_−*M*_(*n*−1)_)/*M*_(*n*)_)^*γ*^, where *M* is the mean body mass of the species index *n* in a fully censused community (Restrepo, Renjifo, & Marples, 1997). Alternatively, Siemann and Brown (1999) log‐transformed the data, and the difference was calculated as SB = log_10_((*M*_(*n*+1)_)/(*M*_(*n*)_)). Although informative, these difference index methods suffer from inherent subjectivity.

Various resampling methods were developed to detect discontinuities more objectively. The Discontinuity Index developed by Stow et al. (2007) tested the vector norm of the observed data against a population of hypothetical Discontinuity Index values. The hypothetical population was created by resampling a hypothetical null distribution of uniform distances with the same sample size as the observed dataset. The result is a single value describing the probability that the Discontinuity Index of the dataset is higher than that created randomly. Straightforward and robust, the Discontinuity Index (Stow et al., 2007) is useful as a general metric describing the dataset but does not identify where the discontinuities are located along the rank‐ordered axis.

The Gap Rarity Index (GRI) (Restrepo et al., 1997) utilized a neutral null model that was repeatedly sampled as: gap(*n*) = log10(*M*_(*n*+1)_)−log10(*M*_(*n*)_), where *M* is the mean body mass of the species *n* in a fully censused community. The resampling creates a hypothetical distribution of gaps against which the gaps within the real data are tested. The GRI has been the most widely used method to date in discontinuity analysis of census data (Nash, Allen, Baricjievy et al., 2014; Sundstrom et al., 2012; Wardwell 2008) and is preferred due to its simplicity of inference. The GRI uses a neutral model, based on a standard kernel density estimator, that is generated to approximate a unimodal continuous distribution and is used to analyze for evidence of multimodality (Silverman, 1981). Practically, this sets up a null hypothesis against which the real data are compared to test for departure from some form of central tendency, which is expected from equilibrium systems (viz. Allen, Forys, & Holling, 1999; Forys & Allen, 2002). Discontinuities are defined as areas between successive, ranked body masses significantly exceeding that generated by the continuous null distribution. This test for departure is carried out using a resampling approach, which allows for robust comparison, incorporates the effects of sample size, and accounts for the associated uncertainty of whether a discontinuity is real or simply a sampling artifact. However, the major limitation of the GRI algorithm is that it uses arbitrary constants which are not a dataset‐specific estimate, but rather a constant used in the original programming which has unclear biological origins and so cannot be rigorously applied across different types of complex systems.

Other statistical approaches are hierarchical cluster analysis (HCA) and classification and regression trees (CART), including the Bayesian version thereof (Chipman, George, & McCulloch, 1998). These three methods use various algorithms to find the “best” clustering based on minimizing a cost function. BCART is notably the most reliable method, where the tree generating algorithm minimizes within‐group entropy, which is consistent with the premise of the discontinuity theory. BCART is relatively robust to variations in tuning, provides repeatable results, and was used successfully to show regime shifts in paleo‐diatom community assemblages (Spanbauer et al., 2016) and the resilience of plankton communities at macroecological scale (Baho et al., 2015).

HCA, CART, and BCART are optimal for clustering and classification of large multivariate datasets; however, these methods are also difficult to interpret in the context of discontinuity analyses. The algorithms essentially partition variance and are not testing any hypothesis or null model. They are useful in partitioning data into subpopulations of data with latent variables; however, we are utilizing univariate data where the datum is a proxy for the scale of pattern and process. Therefore, there is no ecologically meaningful inference that can be made on the results of variance partitioning methods, and so the “significance” of a cluster of body masses is difficult to understand. Stow et al. (2007) have advocated finding consensus using multiple methods. The determination of what constitutes a significant cluster will always be a challenge and remains a contentious issue when identifying the location of discontinuities. Thus, there is a need for a contemporary, intuitive method to detect discontinuities from census data. We present here an application that compares the observed distribution with that generated from a neutral null, formulated from a Gaussian distribution. We then utilize a bootstrapping approach to test to compare the observed data versus that generated from the neutral null to quantitatively assess the likelihood that differences between body masses are generated randomly, or are likely to represent a discontinuity. Although most closely aligned to the GRI as an approach to discontinuity analysis, the method presented here is novel in that it does not utilize any fixed conversion or tuning parameters and therefore can be used across multiple systems in which the user may want to test for discontinuities; it is intuitive to understand the outputs; and it will be publicly available as an R script to allow straightforward application across studies.

## 2 METHODS

Our method, the discontinuity detector (DD), has two components: the development of a neutral null distribution and a bootstrapping approach that compares rank‐ordered differences in data to differences generated through resampling the neutral null. We then compare the observed data to the bootstrapped samples and calculate the percentile of the bootstrap distribution to infer the likelihood that the gap is not through random chance.

### 2.1 Data requirements

The DD is designed for census data, as opposed to sample data. We use body size as an example, but researchers working in other types of complex systems have chosen proxies that represent an integration of system‐specific drivers, such as firm size or city size (Sundstrom et al., 2014) and biomass (Angeler et al., 2012). Abundance measures are not required, just presence data such as the bird or mammal species assemblages found in boreal forests, grasslands, and arid areas as found in Holling (1992). Although our method may be appropriate for determinate growth species, further research is required for indeterminate growth species such as fishes (Nash, Allen, Barichievy et al., 2014).

### 2.2 The neutral null

Kernel density estimates of log_10‐_transformed data (*m*) are generated for bandwidth values (*h*) ranging from 0 to half of the range in the dataset. We used half of the range as this avoids too many edge effects being introduced by the smoothing function and makes the computation more efficient; however, this can be changed if necessary by the user. A lower bound of zero was used as body masses can only be positive so it is not sensible to generate a model that predicts negative body mass. We utilized a bandwidth increment of 0.001 (i.e., *h*_(*j *+ 1)_ = *h*_(*j*)_ + 0.001 for *j* = 1… *n*). The estimates are easily calculated in R using the base package and the density function (Equation (1) and (2)), with the bandwidth bounded to be greater than 0 (0 < *h*<*∞*).

(1) $${\hat{f}}_{\left(h_{j}\right)}\left(m\right)=\frac{1}{nh_{j}}\sum_{\left(i=1\right)}^{n}K\left(\frac{m-m_{i}}{h_{j}}\right),$$

where *K* is the standard normal density function

(2) $$K\left(x\right)=\frac{e^{-\frac{1}{2}x^{2}}}{\sqrt{2\pi }}$$

and *h* is the bandwidth at index *j*, against which the masses of vector *m*, at index *i* are calculated. A kernel density estimator generates, for a given bandwidth, a smoothed curve that integrates to 1, which shows the “density” of data points at the particular value.

The output is a set of smoothed kernel density functions of varying bandwidths ranging from 0 to half of the data range. For each of these kernel density estimates, the second derivative test is utilized to determine whether the function is unimodal. The neutral null model is selected as the kernel density estimate with the smallest bandwidth that is unimodal.

We have used the Gaussian function as the smoothing function as it speaks to the common ecological expectation that there is a limited range of scales of resource distribution, implying an optimum scale at which an animal can exploit resources. For instance, in the absence of reinforcing process rates, we would not expect multiple scales or hierarchical pattern and process. Instead, an equilibrium of pattern and process would be reached (Wu & Loucks, 1995) which would manifest as processes being distributed over a continuous, unimodal distribution within the bounds of realistic system scales. There may be utility in having a uniform distribution as a neutral null model, but the ecological implication of a uniform distribution is that there is no optimum scale at which pattern is more or less likely to occur. Currently, the ecological meaning behind using a uniform distribution as a null hypothesis is not evident, but the method presented does allow for user‐defined neutral nulls to be generated and tested.

### 2.3 Resampling and calculating the percentile

The neutral null represents a null hypothesis of an “optimal” body size for a given system. This neutral model is redrawn without replacement (we used 5,000 times), with the same sample size as the observed data. For instance, if there are 60 species in the community, a sample size of 60 is resampled without replacement 5,000 times. For each draw, the sampled data are rank‐ordered and the differences between each of the rank‐ordered, simulated body masses are calculated. By doing this multiple times, a distribution of gap sizes is generated for each rank‐ordered gap. Each of the observed differences is compared to the resampled distribution of the same rank, and the percentile (the value below which a given percentage of observations in a group of observations fall) of the resampled gap distribution is calculated. The gap percentile is then used as a measure of how likely the observed difference between census points is to those which can be considered randomly sampled from the neutral null distribution. Decisions regarding what percentile to accept as a gap are akin to alpha value assessment in any frequentist method, and inference can be made accordingly.

### 2.4 Comparison of existing methods

To illustrate whether the DD algorithm presented here is consistent with the methods used in the discontinuity literature, we compared the outputs of the DD algorithm to those from the GRI, CART, BCART, and HCA for datasets previously published in discontinuity studies. These datasets are body mass distributions for boreal forest mammals (*N* = 36), boreal forest birds (*N* = 101), boreal prairie mammals (*N* = 53), boreal prairie birds (*N* = 108) (raw data, including home ranges, are available in Holling, 1992), as well as Kalahari mammals and fynbos birds (Allen, 1997).

### 2.5 Sensitivity analysis

The DD must be robust to common research challenges of realistic census sizes (number of species in a community), and the method must be verified for incomplete census data (sample data). We tested three hypotheses: The DD algorithm can reliably detect gaps in data that encompasses (a) multiple scales, (b) under conditions of reduced census success, and (c) under conditions of low species richness.

To approximate realistic ecological scenarios, we simulated a dataset similar in scale and composition to the Holling (1992) boreal bird body mass dataset from which a species pool of 102 birds yielded ten discontinuities which separated 11 clusters of similarly sized birds. Gaussian mixture models were used to generate data of known multimodal distributions across scales of analysis. The multimodal distributions represent the multiple and discrete scale domains from which a sample is drawn. For these sensitivity analyses, test data were drawn from a Gaussian mixture model with ten modes with means and variances increasing evenly on a log scale. A hypothetical census of 100 data points (simulating species average body masses sampled from a multimodal distribution) was generated with equivalent weights in the mixture.

One thousand test datasets with differing sample sizes and census success (ability to detect all species in the community) were generated and tested for discontinuities using the DD. This generated a population of discontinuities from which the effects of sample size and census success could be tested. Because each experimental dataset was an independent simulation, it was necessary to objectively cluster discontinuity values together to measure the effect of changing simulation parameters on the location of discontinuities. If the algorithm reruns an analysis on the same dataset, yet the discontinuities are identified in different places, the algorithm is not robust, by running repeated analysis of the same dataset we explicitly tested for this. To do this, the DD was run on all test datasets, and then, independent estimations of discontinuities were clustered together using the mClust package which uses a Bayes clustering algorithm that selects clusters based on the BIC (Bayes information criteria ~ similar to an Akaike information criteria) value was used to determine the best‐fit number of clusters. The distribution of discontinuities per cluster was then compared under varying conditions of census success and sample size to determine the sensitivity of DD to real‐world constraints in the census:

- The population of discontinuities was compared among samples with equal sample size. Sample success was simulated by randomly deleting a proportion of the sampled population. Comparisons were made between proportions sampled from 0.75 to 1.
- The population of discontinuities was compared among samples with unequal sample size. Sample success is assumed to be 1, with sample sizes varying from 20 to 100 samples (increments of 10 values) in the proxy dataset.

## 3 RESULTS

**Figure 2:**
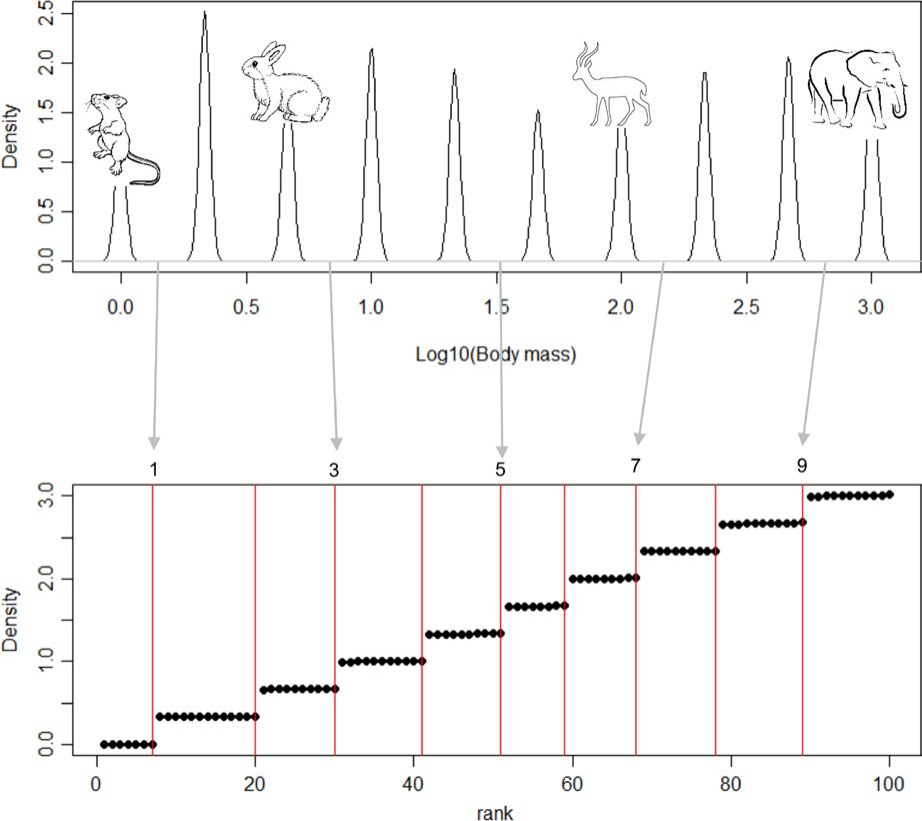
Hypothetical census of 100 species, sampled from 10 equally weighted normal mixture models and analyzed for discontinuities using the discontinuity detector. Each black dot represents a species, while groups of black dots represent clusters of similarly sized species, and clusters are separated by discontinuities shown with a red line. Inset mammal images illustrate the scaled nature of the body mass distributions being simulated by showing an example of a species that would fall in that hypothetical cluster. Arrows point to the discontinuities in the rank‐ordered census data. The five discontinuities indexed here are presented in the sensitivity analysis

**Figure 3:**
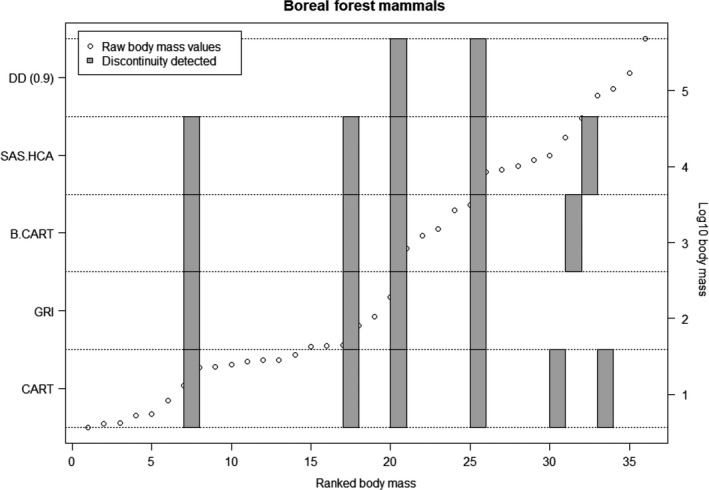
A comparison of the discontinuity detector versus the other methods. The hollow circles represent the raw body mass data (log 10 scale, on right‐hand axis.) against the rank body size (*x*‐axis). The gray squares represent where a discontinuity has been identified, by each of the different methods. Each method is stacked on top of each other to show which rank the discontinuity has been detected (*y*‐axis—left‐hand side). We compare hierarchical cluster analysis (HCA), Bayesian classification and regression trees (BCART), classification and regression trees (CART), and Gap Rarity Index (GRI). The (0.9) represents the acceptance value used to accept the discontinuity, that is, the observed data are >90% percentile of the bootstrap comparison. Here, we present the Boral forest mammal dataset of Holling (1992), in Figure 4, we show the results, presented similarly, of the comparisons for other datasets

**Figure 4:**
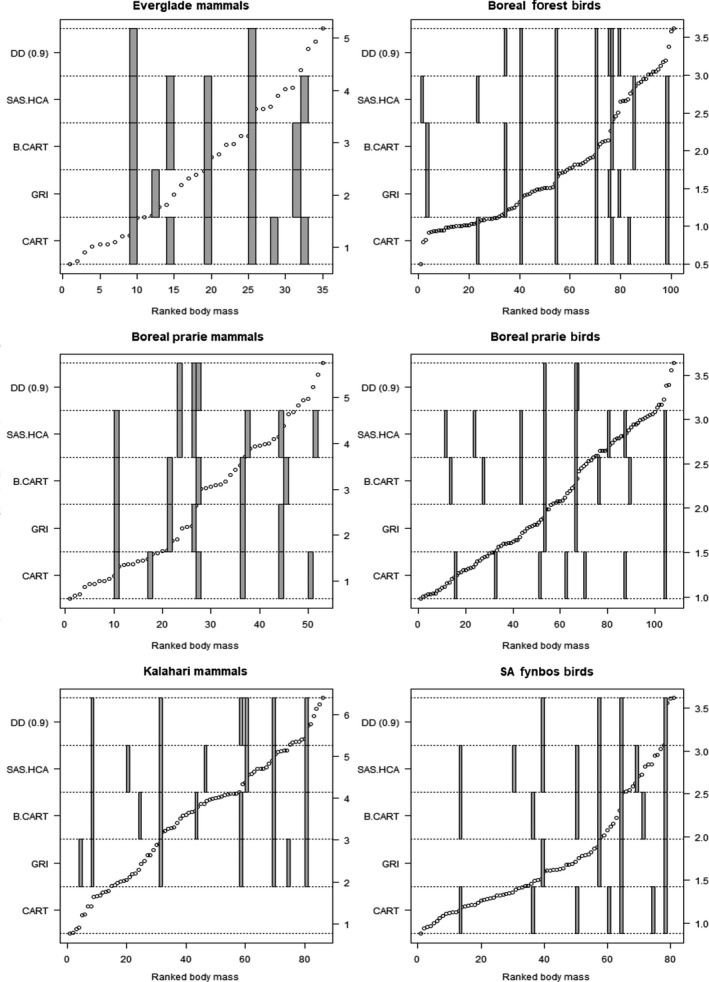
A comparison of the discontinuity detector versus hierarchical cluster analysis (HCA), Bayesian classification and regression trees (BCART), classification and regression trees (CART), and Gap Rarity Index (GRI). Compared for six datasets from Holling (1992) displayed as a subplot. As shown in Figure 3, within each subplot, the hollow circles represent the raw body mass data (log 10 scale, on right‐hand axis.) against the rank body size (*x*‐axis). The gray squares represent where a discontinuity has been identified, by each of the different methods. Each method is stacked on top of each other to show which rank the discontinuity has been detected (*y*‐axis—left‐hand side)

R code for implementing the DD is provided in the Supporting information. The DD method presented in the paper successfully identified known gaps from multimodal mixture models (Figure 2). When comparing the DD results to previous methods (i.e., CART, BCART, HCA, and GRI), we found consistency across many of the methods. This is evident in both the Boreal foret mammals dataset (Figure 3) and other sample datasets (Figure 4, data from Holling, 1992). The similarity between the GRI and the DD is to be expected, as they are fundamentally similar approaches. Both GRI and DD are more conservative than the variance partitioning approaches of HCA, CART, and BCART, identifying fewer discontinuities.

**Figure 5:**
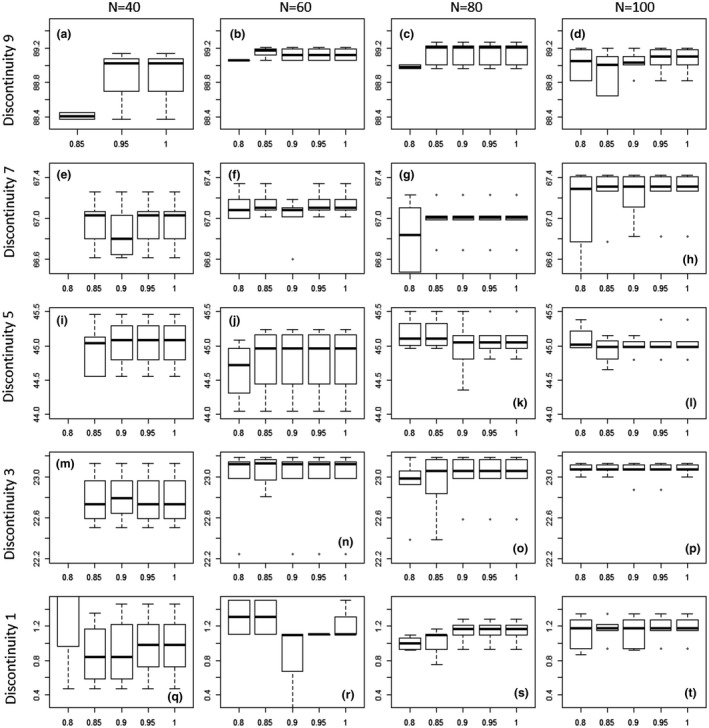
Sensitivity analysis of the discontinuity detector algorithm under conditions of varying sample size and sampling success. The *x*‐axis shows the range of sample sizes. Column 1: a, e, i, m, and q = 40 species, column 2: b‐r = 60 species, column 3: c‐s = 80 species, and column 4: d‐t = 100 species. The *y*‐axis represents five of the discontinuities (number 1, 3, 5, 7, and 9, as generated and shown in Figure 2). Within each subplot, the box and whisker represents the proportional success of the census, so for example, plot Q has 40 species, and shows the consistency of the DD when 80%, 85%, 90%, 95%, and 100% of species are successfully sampled (i.e., how robust is the method to missing species or data in the census)

The method detects discontinuities accurately and precisely under increased sample sizes and census success (Figure 5, top right); however, under relatively lower sample sizes and low census success (Figure 5 bottom left), there is less precision and accuracy in the location of the discontinuities.

## 4 DISCUSSION

The discontinuity detector provides an objective, repeatable method to find discontinuities in census data. This method provides an easy approach for determining scales in research studies which has great utility for complex system‐centered research such as predicting invasion and extinction, evaluating ecosystem function across scales, and assessing system resilience (Allen & Holling, 2008; Allen et al., 1999, 2005; Sundstrom et al., 2012). The method may, therefore, be useful for addressing management challenges that derive from environmental change (Angeler et al., 2016).

The DD method presented here improves on the original GRI method by removing a subjective transformation constant, which makes inference simpler and the method more transparent. The use of the neutral null allows for a hypothesis testing approach as opposed to simply the clustering approach used in alternative methods, which makes for stronger inference around the nature of the discontinuity.

We should also note that while it is unlikely that the method will miss gaps that are in fact real, it is sensitive to detecting erroneous gaps. Accordingly, interpretation of the results of the DD must be made considering type 2 errors. Overall, the DD algorithm is more conservative than the variance partition methods (HCA, CART, BCART), which is a result of comparing the observed data to a unimodal neutral null (Figure 3). A major challenge in detecting discontinuities in census data is that there exists no unambiguous null model or standard for comparison. Standard statistical procedures arise from trying to make a population‐level inference from a (proportionally) small sample. In the investigation of discontinuities from census data, one is making inference on an extremely small population from a very large (proportional) sample; hence, the concepts from traditional statistical investigations are not fully transferable. We utilize a null hypothesis of a continuous unimodal distribution to simulate a central tendency. The method presented here does allow for varying null models to be used; however, any null model that is used as a basis for comparison can only ever be hypothetical, which opens methodological questions surrounding the null model choice as opposed to the discontinuities themselves. Therefore, the ecological meaning behind a uniform null or skewed null model, for example, would need to be defined prior to the test.

How well the census represents what's in the community and how many species are in a community will affect the precision of the DD in detecting the discontinuities. This is because the variance in the location of the resampled distribution is a function of the number of samples from which the resampling is drawn. If only ten samples are taken from a distribution, the variability in the gaps between individual samples is higher than when there are around 100 samples. Practically, this may limit the application of the DD for species‐poor systems (such as a desert) but, if the census largely accounts for the species in the community, the method will be effective in detecting discontinuities.

The DD method provides a quick, easy to use and, most importantly, transparent method to objectively detect discontinuities in census data. Such a method will allow for increased discontinuity research in the various fields of ecology and other complex system science affording the ability to build upon this burgeoning area of study.

## CONFLICT OF INTEREST

None declared.

## AUTHORS’ CONTRIBUTION

CB was the primary author. CB and KN tested the application. DA, TE, AG, KN, CS, SS, and CA provided development discussion, critique of the method, and contributed significantly to the writing of the manuscript. CA provided the source code for original Neutral Null and obtained permission from the author and helped to develop the theory behind the method.

## DATA ACCESSIBILITY

All data used are freely available from Holling (1992): https://esajournals.onlinelibrary.wiley.com/doi/abs/10.2307/2937313. Source code supplied as Supporting information in this manuscript.
