# Supplementary material for: A method to detect discontinuities in census data
Source: Ecol Evol. 2018 Sep 20;8(19):9614–23. doi: 10.1002/ece3.4297 (PMC6202717; doi:10.1002/ece3.4297)
Supplement: Supplementary file 1 [file ECE3-8-9614-s001.docx]

#Supporting data for the manuscript entitled:

**A method to detect discontinuities in census data**

#Authors: Chris Barichievy*, David G. Angeler, Tarsha Eason, Ahjond S. Garmestani, Kirsty L. Nash, #Craig A. Stow, Shana Sundstrom, and Craig R. Allen.

#Coded May 2017

#Contact: Chris Barichievy cbarichievy@gmail.com / Chris. Barichievy@zsl.org

#----

# The Discontinuity Detector ( DD) calls 2 functions:

# The neutral Null and The Discontinuity Detector

#hnull<-Neutral.Null(log10.data,resolution)

#Bootstrap.gaps<-DD(log10.data,hnull,Sample.N)

#Defaults are set to mimic the original code used in Restrepo 1997

#1.Neutral.Null

Neutral.Null<-function(log10.data,resolution=4000){

Dmax=max(log10.data,na.rm=FALSE)

Dmin=min(log10.data,na.rm=FALSE)

ds=(Dmax-Dmin)/resolution

MaxK=(Dmax-Dmin)/2

MinK=ds*2

#define h's to analyze

ks=seq(MinK,MaxK, by=1/resolution)

#generate matrix

bws=matrix(data=NA,nrow=length(ks),ncol=1)

for(i in c(1:length(ks))){

#calculate KS density estimate

KSdens<-density(log10.data,bw=ks[i],"gaussian", adjust=1)

#Test if the ksdensity is unimodal

TF<-which(diff(sign(diff(KSdens$y)))==2)+1

if (length(TF)==0)bws[i]=1

else bws[i]=0

}

#Define the neutral Null

r=min(which(bws==1))

hnull=ks[r]

return(hnull)

}

#2. bootstrap function

DD<-function(log10.data,hnull, Sample.N=1000){

NNull<-density(log10.data,bw=hnull,"gaussian", adjust=1)

N<-length(log10.data)

#generate matrix

null.samples<-matrix(data=0,ncol=Sample.N, nrow=N)

for(i in 1:Sample.N){

#sample the null model

rand.N<-sample(NNull$x, N, replace=TRUE,prob=NNull$y)

#calculate the gaps

null.samples[,i]<-sort(rand.N,decreasing=FALSE)

#put into the matrix

}

#generate gaps

gaps.log10.data<-diff(log10.data)

gaps.null.samples<-diff(null.samples, decreasing=FALSE)

gap.percentile<-matrix(data=0,nrow=length(gaps.log10.data),ncol=1)

for(i in 1:length(gaps.log10.data)){

#generate distribution of gaps per row (per gap rank)

gap.percentile[i]<-ecdf(gaps.null.samples[i,])(gaps.log10.data[i])

}

Bootstrap.gaps<-rbind(gap.percentile,0)

Bootstrap.gaps<-cbind(log10.data,Bootstrap.gaps)

return(Bootstrap.gaps)

}

#call

hnull<-Neutral.Null(log10.data,resolution)

Bootstrap.gaps<-DD(log10.data,hnull,Sample.N)
